# Experience Feedback Committee: a management tool to improve patient safety in mental health

**DOI:** 10.1186/s12991-015-0062-2

**Published:** 2015-09-03

**Authors:** Bastien Boussat, Thierry Bougerol, Olivier Detante, Arnaud Seigneurin, Patrice François

**Affiliations:** Public Health Department, Grenoble University Hospital, Grenoble, France; Laboratory TIMC, UMR 5525, CNRS, Joseph Fourier University, Grenoble, France; Psychiatry Department, Grenoble University Hospital, Grenoble, France; Neurology Department, Grenoble University Hospital, Grenoble, France

**Keywords:** Risk management, Mental health, Neuropsychiatry, Quality improvement, Interdisciplinary communication

## Abstract

**Background:**

A management tool, called the Experience Feedback Committee, has been applied for patient safety and successfully used in medical departments. The purpose of this study was to analyse the functioning of an Experience Feedback Committee in a psychiatric department and to explore its contribution to the particular issues of patient safety in mental health.

**Methods:**

We conducted a descriptive study based on all the written documents produced by the Experience Feedback Committee between March 2010 and January 2013. The study was conducted in Grenoble University Hospital in France. We analysed all reported incidents, reports of meetings and event analysis reports. Adverse events were classified according to the Conceptual Framework for the International Classification for Patient Safety.

**Results:**

A total of 30 meetings were attended by 22 professionals including seven physicians and 12 paramedical practitioners. We identified 475 incidents reported to the Experience Feedback Committee. Most of them (92 %) had no medical consequence for the patient. Eleven incidents were investigated with an analysis method inspired by civil aviation security systems. Twenty-one corrective actions were set up, including eight responses to the specific problems of a mental health unit, such as training to respond to situations of violence or management of suicide attempts.

**Conclusions:**

The Experience Feedback Committee makes it possible to involve mental healthcare professionals directly in safety management. This tool seems appropriate to manage specific patient safety issues in mental health.

**Electronic supplementary material:**

The online version of this article (doi:10.1186/s12991-015-0062-2) contains supplementary material, which is available to authorized users.

## Background

Patient safety has become a public health priority in the past 15 years since the publication of major epidemiological studies on healthcare-related adverse events [1–5]. The last Canadian national study estimated that 7.5 adverse events occurred for 100 hospital admissions, including a high proportion of preventable events and adverse events leading to death [6].

Even if many adverse events are similar in all medical units, there are specific patient safety issues in mental health. Indeed, neuropsychiatric units face events caused by behavioural problems such as violence, absconding, self-harm and suicide attempts [7–11]. Mental health adverse events result from multiple factors, mixing human behaviour risks and healthcare organizational weaknesses. Considering the lack of readily available information to guide patient safety systems in mental health, the improvement of management tools is essential to promote a patient safety culture among healthcare professionals [11–13].

Since the 1970s, civil aviation has developed operating experience feedback to improve passenger safety. Air transport safety systems require that any incident, even minor, must be treated by a systemic analysis within the air crew. Inspired by those security systems, a specific mechanism, called the Experience Feedback Committee (EFC), was created in 2005 to analyse adverse events within a medical team. In France, the method was adapted to healthcare facilities with the help of Air France Consulting and was successfully implemented in an emergency department and a radiotherapy unit [14–19]. The EFC is a team composed of professionals representing the diversity of the functions encountered in the medical unit. The EFC members meet monthly to examine adverse events reported to their medical unit. An event requiring a thorough analysis is chosen at each meeting according to its severity and frequency and corrective actions are suggested based on the results of the analysis. The main principles of the method are managing patient safety within a medical team and setting up corrective actions concerning latent factors that contributed to the occurrence of events or near-miss events.

The aim of this study was to describe the functioning of the EFC in a neuropsychiatric department and to discuss its contribution to the management of patient safety.

## Methods

### Study design

We conducted a descriptive study based on the written reports of the neuropsychiatric department EFC from its beginning in March 2010 until January 2013.

### Setting

The study was conducted in a 1347-bed acute-care university hospital. The neuropsychiatry department has an annual patient volume of 4800 stays.

Adverse events and near-misses affecting patients (such as nosocomial infections, technical complications, negligence, diagnostic mishaps, therapeutic incidents, etc.) are reported by healthcare professionals, through a voluntary internal reporting system, to the hospital’s central safety unit using a standardized report form. This unit is composed of a medical doctor, a pharmacist and an engineer specializing in quality management. The reports of events classified by severity and risk areas are presented during a weekly meeting involving representatives of the administration and professionals in charge of specific risk areas such as the risks associated with drugs (pharmacovigilance), nosocomial infections (infection vigilance), healthcare materials and devices (medical device vigilance), transfusion (haemovigilance), etc. The central safety unit directly investigates the most serious events and those involving several hospital departments. Other events are transmitted to the appropriate operator and to executives of relevant departments. For departments where an EFC has been implemented, the central safety unit addresses the reports of events to the EFC leader every month.

### Neuropsychiatric department EFC

The neuropsychiatric department EFC was set up in March 2010 and works through a written procedure in accordance with the method proposed by Air France Consulting [17, 18, 20]. The Committee is composed of volunteer representatives of the various professions within the neuropsychiatric division. A few days before the committee meeting, the EFC leader receives a file with event reports concerning the neuropsychiatric division. Committee meetings are conducted according to a standardized framework: (1) reading the list of reported events, (2) choosing a priority event to investigate by consensus according to the criticality of each incident, (3) choosing the professional responsible for the investigation, (4) reviewing the analysis made of the event chosen the previous month, (5) choosing corrective actions and (6) monitoring on-going actions. The investigation is carried out during the month following the EFC by a designated person using a method, called ORION©, developed from methods of systemic analysis used in civil aviation and adapted to the healthcare domain by Air France Consulting [14–17]. Previously trained investigators must follow the main steps of the ORION© method to fill out a standardized report (Additional file 1: Appendix): collecting data and existing recommendations, describing the chronological facts that occurred before, during and after the event, describing the failures, looking for causes of errors and latent factors that could have contributed to the failures, setting up corrective actions and writing a report of the analysis. Causes and latent factors are sought in different areas such as political, organizational, working conditions, team functioning, procedures, actors and the patient.

### Data collection

All written documents from the EFC of the neuropsychiatric division were analysed. The events reported were classified according to the source of the report, the type of event and the consequence for the patient using the International Classification for Patient Safety [21]. Written reports from meetings were analysed using a standardized form that included the theoretical steps of an EFC meeting and the contents of the ORION© analysis (as described above). All documents were analysed by two independent investigators. Differences in rating were discussed until a consensus was reached.

### Statistical analysis

We reported the characteristics of the EFC’s main functioning (meetings and participants), the adverse events reported and the analysis reports as medians and interquartile ranges (IQR; i.e., 25th and 75th percentiles) for continuous variables and number and percentages for categorical variables. The analysis was performed using R version 3.0.1.

## Results

The committee set up 30 meetings during the study period. A total of 22 professionals participated in the EFC (Table 1), including seven physicians, four head nurses, four auxiliary nurses, three nurses, one secretary, one physical therapist, one cleaning staff member and one quality engineer. The median number of attendants was eight (IQR 6–9) per meeting. A report was written for each meeting. Priority events were chosen in half of the meetings, analysis reports were presented and corrective actions were decided in more than one-third of the meetings (Table 1). The previous corrective actions were monitored in 19 meetings (63.3 %).

**Table 1:** Main functioning characteristics of the Experience Feedback Committee of the neuropsychiatry department

	*N* = 22	%
Participants		
Physicians	7	31.8
Head nurses	4	18.2
Auxiliary nurses	4	18.2
Nurses	3	13.6
Secretary	1	4.5
Quality engineer	1	4.5
Physical therapist	1	4.5
Cleaning staff member	1	4.5
Median number of participations per participant (IQR 25–75)	9	(5–15)
Median number of participants per meeting (IQR 25–75)	8	(6–9)

	*N* = 30	%
Meetings		
Writing of minutes	30	100.0
Listening to the events reported during the previous month	30	100.0
Choosing a priority event to analyse during the following month	15	50.0
Listening to the analysis report from the event chosen the previous month	11	36.7
Deciding corrective actions	11	36.7
Following up the previous corrective actions	19	63.3

A total of 475 reported incidents were transmitted to the EFC (Table 2). A median number of 12 incidents (IQR 7–20) were discussed per meeting. Incidents were mainly (97.1 %) reported by a professional of the department and 93.3 % of them occurred inside the department. Reported incidents concerned mainly clinical administration (29.3 %) (including incidents in patient identification, patient transfer, admission, discharge), behaviour (24 %) (concerning patient or staff) and patient accidents (12 %). The majority of incidents had no clinical consequence for the patient (91.8 %) or the care process (70.1 %). In 29 cases (6.1 %), the reported event had a mild or moderate consequence for the patient (Table 2). Among the 20 events involving a mild consequence for the patient, ten events were related to a patient fall, four events concerned violence against the staff or another patient, three events were related to inadequate equipment and three events were suicide attempts. Among the nine events involving a moderate consequence, eight falls led to orthopaedic fracture or head trauma, and one admission error concerned a patient hospitalized in a corridor.

**Table 2:** Characteristics of the events reported during the Experience Feedback Committee meetings

Characteristics	*N* = 475	%
Incident type		
Clinical administration	139	29.3
Behaviour	114	24.0
Patient accidents	57	12.0
Infrastructure/building/fixtures	41	8.6
Medical device/equipment	30	6.3
Resources/organizational management	30	6.3
Nutrition	20	4.2
Clinical process/procedure	16	3.4
Medication/IV fluids	14	2.9
Documentation	11	2.3
Healthcare-associated infection	3	0.6
Blood/blood products	0	0.0
Oxygen/gas/vapour	0	0.0
Degree of Harm		
None, without care modification	343	72.2
None, with care modification	103	21.7
Mild	20	4.2
Moderate	9	1.9
Severe	0	0.0
Death	0	0.0
Report provider		
Staff from the neuropsychiatric department	461	97.1
Staff from another department	16	3.4
Place of the event		
In the neuropsychiatric department	443	93.3
In another department	32	6.7

Fifteen priority incidents were chosen for investigation, including six incidents related to clinical administration, four incidents related to behaviour problems, four incidents related to infrastructure and fixtures and one to the keeping of archives. Four incidents chosen were not investigated. Among the 11 analyses carried out, nine reports were written, whereas two reports were only oral (Table 3). Three reports involved a problem of coordination with other hospital departments. First, an error of emergency transfer was reported for a patient hospitalized in a corridor who was transported to the intensive care unit for optimal surveillance because of loss of consciousness. Second, stretchers for an imaging examination emergency were recurrently unavailable. Third, organizational problems of the psychiatric consultation were reported. Three reports analysed adverse events associated with behavioural disorders: the inability of a professional to properly manage a patient with a suicide risk in child psychiatry, an attempted suicide by strangulation with a phone cord and finally the investigation of repeated fugues in adult psychiatry. Three ORION© analyses were carried out to investigate a technical equipment failure including an ECG machine, the computer network and a power failure. The last reports concerned a loss of patient records, a delivery mistake for special meals and the fall of an elderly patient.

**Table 3:** Characteristics of the analysis reports and of the corrective actions

	*N* = 11	%
Analysis reports		
Written reports	9	81.8
Oral reports	2	18.2
Description of the data collection method	9	81.8
Individual interviews	9	81.8
Collective debriefing	1	9.1
Files	5	45.5
Area visits	6	54.5
Description of the chronology of facts	9	81.8
Description of existing recommendations	6	54.5
Error identification	6	54.5
Identification of contributing or latent factors	10	90.9
Management	5	45.5
Organization and procedures	7	63.6
Working environment	6	54.5
Teamwork	3	27.3
Technical processes	5	45.5
Professionals	4	36.4
Patients	2	18.2
Corrective actions		
Proposed actions	*N* = 26	
Staff Training	4	15.4
Writing procedures	12	46.1
Organizational changes	4	15.4
Increasing material resources	5	19.2
Decided actions	*N* = 21	
With a professional in charge	14	66.7
From the department	12	57.1
From another department	2	9.5
With a defined deadline	9	42.9

The expertise of the written reports showed that the ORION© method was frequently followed. The chronology of the facts and the identification of contributing or latent factors were described in 80 % of the cases. However, existing recommendations were only described in one-half of the cases. Twenty-six corrective actions were proposed by the professionals who performed the analyses. The committee decided to implement 21 actions. Written guidelines (*n* = 11) were the most common type of action (see box). Other actions included staff training, improvement of the availability of material resources and a deeper analysis of an event in the context of a medical thesis. For example, the ORION© report related to the fall of an elderly patient showed several factors contributing to the event: some factors related to the patient (inappropriate behaviour of a patient with a depressive syndrome, decreased alertness and reflexes due to anxiolytic treatment) and organizational factors (lack of assessment of the risk for falls at admission, lack of supervision due to a high level of department activity). The corrective actions selected were structured around these two main types of factors. A systematic screening of risk factors for falling at admission was established. Secondary prevention actions were also decided: pharmaceutical adaptation to reduce iatrogenic events and implementation of monitoring for high-risk patients by a team composed of a physiotherapist, an occupational therapist and a movement therapist. Finally, adjustments were made at the facility level with adjustable-height beds, night lights in the rooms and more convenient showers.

**Box:** Corrective actions set up

Guideline writing
Inpatient transfer from ED
Patient medical record management
Suicide risk assessment at admission
Management of patient with behaviour problems
Security guards system
Job profile of secretary
Failing risk assessment at admission
Protocol to prevent failing risk
User manual for ECG advise
Emergency consultation procedure
Electrical failure procedure
Training
Respond to situations of violence
Management of elderly patients
Respond to suicide attempt
Proper management of medical records
Material resources
Establishment of an isolation room
Change of ECG device
Loan of two ECG device
Implementation of a planning software for inpatient transport
Research
Completion of a medical thesis on inpatient transfers in the hospital

## Discussion

This study highlighted that the EFC implemented in the mental health department functions routinely with patient safety incidents analysed and corrective actions set up. The EFC method, which was successfully implemented in medical units, is also relevant to managing patient safety in mental health. Nath and Marcus demonstrated that some patient safety incidents and contributing factors are specific to mental health [11]. Although the EFC examined events that might have been found in other medical units, events specific to mental health were also reported and analysed. For example, suicide attempts and missing person incidents were investigated and the analyses highlighted several organizational flaws in suicide risk assessment, monitoring patients at risk and securing the department’s premises. Consequently, actions concerning the security of the building and the assessment of suicide risk in child psychiatry (specific staff training to manage patients with violent behaviour and improved guidelines) were set up.

Considering patient safety incidents in mental health as a result of a complex set of contributing factors, the EFC provided a structured framework to analyse them within the department’s routine [8, 13, 22, 23]. The principle is to choose only one event per meeting to perform a thorough analysis using the ORION© method. As advocated by the Reason model, this method aims to identify factors related to the design of the system’s organization or the workplace environment rather than individual error [24]. The method implies the main steps of the Association of Litigation and Risk Management (ALARM) protocol but seems easier to use for healthcare professionals who are not specialists in risk management [25]. The essential contribution of an EFC is to provide a formal framework to correct the latent failures in the department’s organization.

Over the 3 years studied, the theoretical framework for conducting an EFC was not always followed. Indeed, the analysis of events did not always include all the steps defined in the ORION© method and the search for contributing factors was often superficial. These deviations can be partly explained by the staff’s lack of time and availability. Carrying out the investigations to determine the causes of events as well as writing the report is time-consuming. Inconsistent monitoring of the corrective actions previously set up resulted in the resurgence of certain patient safety incidents that had previously been investigated, highlighting the importance of monitoring corrective actions by the EFC. A low level of expertise in conducting the analyses may also explain these deviations: only professionals involved in the EFC at its beginning were given formal training, and additional training was not proposed afterwards. Consequently, regular training seems necessary to ensure the quality of meetings and event analysis.

To function properly, the EFC requires reports of adverse events experienced by professionals. Several studies have shown that healthcare professionals, particularly physicians, agree with the importance of incident reporting and the concept of learning from errors [26, 27]. Nevertheless, in practice, many incidents are not reported [28, 29]. Self-report of patient safety incidents is hindered by several barriers such as time constraints, complex forms, fear of punishment, shame as well as lack of education and feedback [28, 30–33]. In the present study, we were not able to estimate the proportion of unreported incidents. However, the committee had enough incidents to discuss every month. Mental healthcare professionals are probably informed more easily of the corrective actions set up and can observe their effects considering that reported incidents are analysed by professionals in the department. Consequently, the existence of an EFC in a mental health department may improve incident reporting.

Psychiatric units provide global and coordinated care for patients through the involvement of many professionals. However, Priest and Borella [34] showed a higher risk of incidents due to the increasing distribution of patient care over multiple practitioners. One of the strengths of the EFC is to gather all categories of professionals working in the unit and reinforcing interprofessional collaboration and promoting teamwork. This multidisciplinary approach also contributes to identifying system vulnerabilities more easily [35].

This study had several limitations. First, the functioning of an EFC depends on the professionals involved and the study was conducted in only one department. Second, the reporting system based on self-reporting by healthcare professionals did not provide the proportion of unreported incidents and did not take into account the patient’s complaints that were treated by the hospital’s legal department. Consequently, we were not able to measure the impact of the EFC on the completeness of incident reports and more generally on the prevalence of adverse events. Third, the impact of the EFC on patient safety was not assessed using clinical outcomes. However, we assumed that corrective actions against identified vulnerabilities resulted in an improvement of patient safety, including an improvement in the patient safety culture among professionals attending the committee.

## Conclusions

The EFC is a tool allowing the direct involvement of mental health professionals to manage patient safety. This innovative management tool is adapted to the specific adverse events encountered in mental health. The theoretical framework for conducting an EFC was not always followed, suggesting the need for simplifying the method for professionals subject to tight time constraints.

## Additional file

Additional file 1:
**Appendix.** ORION standardized report
