# Supplementary material for: Experience Feedback Committee: a management tool to improve patient safety in mental health
Source: Ann Gen Psychiatry. 2015 Sep 3;14:23. doi: 10.1186/s12991-015-0062-2 (PMC4559211; doi:10.1186/s12991-015-0062-2)
Supplement: Additional file 1: — Appendix. ORION standardized report [file 12991_2015_62_MOESM1_ESM.doc]

**Appendix. ORION standardized report**

ORION analysis report

Experience Feedback Committee:

Investigator:

Date:

**I – Incident identification: Data collection**

**Event title:**

**Event date:**

**Event site:**

**Brief description of the event:**

**Event consequences:**

**Modalities for data collection:**

Individual interview  Debriefing  Consultation of medical files  Site visit

**II – Schedule of facts**

*Present facts in the order that they occurred and identify points of failure*

| **Chronological description** | **Guidelines and operational procedures** | **Points of failure, deviations from guidelines** |
| --- | --- | --- |
| 1. **Before the event** | 1. **Before the event** | 1. **Before the event** |
| 1. **During the event** | 1. **During the event** | 1. **During the event** |
| 1. **After the event** | 1. **After the event** | 1. **After the event** |

**III – Root-cause analysis**

*Report the causes and the latent factor for each domain*

1. *Hospital policy*
2. *Organization*
3. *Working conditions*
4. *Team functioning*
5. *Operational procedures*
6. *Healthcare professionals*
7. *Patient*
8. *Summary of failures and causes*

**IV – Proposal for corrective actions**

*Action plan proposed to the EFC to rectify the root causes.*

*Present the possible corrective actions, if possible by indicating efficiency criteria, the professional in charge of implementing the proposal, the planned deadline, an estimate of the cost, the opportunity for scaling up the action).*

| Corrective actions | Efficiency criteria | Deadline | Professional in charge |
| --- | --- | --- | --- |
|  |  |  |  |
|  |  |  |  |
|  |  |  |  |
|  |  |  |  |
|  |  |  |  |
|  |  |  |  |
|  |  |  |  |
|  |  |  |  |

**Analysis report validation during the EFC:**

**EFC members’ comments:**
